# Chlorine cycling and the fate of Cl in terrestrial environments

**DOI:** 10.1007/s11356-020-12144-6

**Published:** 2021-01-05

**Authors:** Teresia Svensson, Henrik Kylin, Malin Montelius, Per Sandén, David Bastviken

**Affiliations:** 1 Department of Thematic Studies – Environmental Change, Linköping University, SE-581 83 Linkoping, Sweden; 2 Research Unit: Environmental Sciences and Management, North-West University, Potchefstroom, South Africa; 3 Swedish Geotechnical Institute (SGI), SE-581 93 Linkoping, Sweden

**Keywords:** Chlorine biogeochemical cycle, Chloride, chlorinated organic compounds, Organochlorines, Soil, ^36^Cl, AOX, Chloroform, Methyl chloride, Hydrological tracer

## Abstract

Chlorine (Cl) in the terrestrial environment is of interest from multiple perspectives, including the use of chloride as a tracer for water flow and contaminant transport, organochlorine pollutants, Cl cycling, radioactive waste (radioecology; ^36^Cl is of large concern) and plant science (Cl as essential element for living plants). During the past decades, there has been a rapid development towards improved understanding of the terrestrial Cl cycle. There is a ubiquitous and extensive natural chlorination of organic matter in terrestrial ecosystems where naturally formed chlorinated organic compounds (Cl_org_) in soil frequently exceed the abundance of chloride. Chloride dominates import and export from terrestrial ecosystems while soil Cl_org_ and biomass Cl can dominate the standing stock Cl. This has important implications for Cl transport, as chloride will enter the Cl pools resulting in prolonged residence times. Clearly, these pools must be considered separately in future monitoring programs addressing Cl cycling. Moreover, there are indications that (1) large amounts of Cl can accumulate in biomass, in some cases representing the main Cl pool; (2) emissions of volatile organic chlorines could be a significant export pathway of Cl and (3) that there is a production of Cl_org_ in tissues of, e.g. plants and animals and that Cl can accumulate as, e.g. chlorinated fatty acids in organisms. Yet, data focusing on ecosystem perspectives and combined spatiotemporal variability regarding various Cl pools are still scarce, and the processes and ecological roles of the extensive biological Cl cycling are still poorly understood.

## Introduction

Chlorine (Cl) is one of the 20 most abundant elements on earth and has various essential functions for living organisms. Chloride, the only stable ionic form of Cl, is the major anion in blood and is present at concentrations of approximately 100 mmol L^−1^ in plasma and interstitial fluid (Yunos et al. 2010). Chloride participates in osmoregulation of cells (White and Broadley 2001) and is as an important electrolyte for regulation of muscle function and synaptic transmission in the neural system. It is also an essential co-factor in enzymes involved in photosynthesis, e.g. PSII photosystem oxidation of water (Winterton 2000). Hence, Cl is a critical plant nutrient and a minimum requirement of Cl for crops of 1 g kg^−1^ dry mass (d.m.) is indicated (White and Broadley 2001).

Many of the most debated organic contaminants, including the well-known persistent organic pollutants (POPs), are chlorinated (Godduhn and Duffy 2003). Although natural halogenated organic compounds have been known since the late nineteenth century (Gribble 2010), this was not widely recognised by environmental chemists, and the dominating view until recently was that chlorinated organic compounds (Cl_org_) in the environment were primarily anthropogenic and often toxic. It is now evident that there is a large production of natural Cl_org_. Nearly 5000 natural Cl_org_ have been identified; they are produced by fungi, lichens, plants, marine organisms, insects and vertebrates including humans (Gribble 2003, 2010, 2015; Öberg 2002). Some specific Cl_org_ have well known physiological functions, e.g. several important antibiotics such as vancomycin. Others have important environment effects, e.g. volatile Cl_org_ (VOCls) that contribute to atmospheric ozone destruction (Winterton 2000). However, the ecological functions of most Cl_org_ and the reasons for their production are largely unknown.

Cl is central in hydrological research as Cl^−^, the globally dominating chlorine species, is highly soluble in water, and oceanic water has a high enrichment factor compared with riverine water; seawater concentrations are in the order of 2500 times larger than freshwater concentrations (Winterton 2000). At a first glance, this has been interpreted to indicate that Cl^−^ is unreactive in the environment, which until recently has been the prevailing view (White and Broadley 2001). Accordingly, Cl^−^ has been seen as a suitable and inexpensive tracer of soil and ground water movements (Herczeg and Leaney 2011; Hruska et al. 2012; Cartwright et al. 2017), and studies using Cl^−^ as a tracer have been the foundation for contaminant transport models (Kirchner et al. 2000). However, as discussed below, there is now clear evidence that in some environments, Cl^−^ is more active than previously thought.

Chlorine-36, ^36^Cl, is a radioactive isotope with a half-life of 3.01 × 10^5^ years and has attracted interest because of its presence in waste from nuclear facilities (Tanaka and Thiry 2020; van den Hoof and Thiry 2012). The long half-life, high mobility in the pedosphere and the potential for substantial biological uptake creates a need for long-term risk assessments related to handling and storage of radioactive waste (Limer et al. 2009). A growing awareness of the complex cycling of Cl in terrestrial environments necessitates a re-evaluation of risk assessments based on the previous assumptions that ^36^Cl in soils primarily occur as ^36^Cl^−^ and is highly soluble and unreactive. This has been highlighted in for instance agricultural soil-plant systems (Le Dizès and Gonze 2019).

During the past decades, there have been several unexpected discoveries regarding the terrestrial chlorine (Cl) cycle. Experiments with ^36^Cl as tracer have confirmed natural chlorination rates corresponding to as much as 50–300% of the annual wet deposition of Cl in several types of soils (Bastviken et al. 2009). Substantial chlorination of organic matter occurs in all studied types of temperate and boreal soils in agricultural and forest areas (Gustavsson et al. 2012; Redon et al. 2013). Moreover, extensive accumulation of Cl_org_ over 30 years in forest soils was recently demonstrated at the ecosystem level (Montelius et al. 2015). In addition, simultaneous and rapid dechlorination of Cl_org_ in soils was also confirmed (Montelius et al. 2016). Enzymatic control of chlorination processes has been described (van Pee and Unversucht 2003; Bastviken et al. 2009; Wever and Barnett 2017), and the genetic capacity to carry out chlorination is widespread among prokaryotes and eukaryotes alike (Bengtson et al. 2009; Bengtson et al. 2013; Weigold et al. 2016). The extensive natural chlorination processes in soil suggest that the Cl turnover likely is linked to common ecosystem processes. Indeed, chlorination rates were recently linked to microbial activity (Svensson et al. 2017), but the fundamental reasons for the extensive soil Cl-cycling are still unclear. A recent review discusses the microbial metabolism of Cl_org_ and the possible links between chlorinating and dechlorinating microbes (Atashgahi et al. 2018).

Several aspects of Cl have been summarised previously, e.g., physiological roles (White and Broadley 2001; Yunos et al. 2010), and the POP perspectives (Winterton 2000; Bidleman et al. 2019) and will not be the primary focus here. Rather, this review represents an update and supplement to previous reviews focusing on the terrestrial Cl cycling (Clarke et al. 2009; Graedel and Keene 1996; Öberg 2002; Leri and Myneni 2010; Öberg and Bastviken 2012; Vodyanitskii and Makarov 2017) called upon by the emerging interest in the behaviour of ^36^Cl in soils and terrestrial ecosystems (Limer et al. 2009; Sheppard et al. 1996; van den Hoof and Thiry 2012; Le Dizès and Gonze 2019; Tanaka and Thiry 2020). Here, we provide an overview of current knowledge on the Cl cycle and the fate of Cl in terrestrial environments. We highlight the latest findings on the occurrence of chloride and Cl_org_ and suggested translocations and transformation processes related with suggested ecosystem Cl budgets. Finally, we discuss knowledge gaps and possible future research directions towards improved understanding of Cl-dynamics in terrestrial environments.

## Fundamental chemical aspects of Cl

Cl, having the atomic number 17, belongs to the halogen group in the periodic table. Molecular Cl is a strong oxidant due to the high electron affinity and electronegativity of Cl. Consequently, molecular Cl is rare in nature, and Cl^−^, which is highly soluble in water, typically dominates in the hydrosphere and in minerals, but, as discussed below, not necessarily in soil.

### Cl isotopes and sources of ^36^Cl

In nature, Cl occurs primarily as the two stable isotopes ^35^Cl (ca. 76%) and ^37^Cl (ca. 24%). In addition, seven radioactive isotopes exist of which six have half-lives of less than 1 h and are of less interest with respect to Cl cycling in the environment. In contrast, ^36^Cl has a half-life of 3.01 10^5^ years and decays with a maximum energy of 709.6 keV either by emitting a beta particle (98.1%) or by electron capture (1.9%) resulting in the end products argon-36 (^36^Ar) and sulphur-36 (^36^S), respectively (Peterson et al. 2007; Rodriguez et al. 2006).

In the environment, ^36^Cl is produced by natural nuclear reactions; in the atmosphere by the spallation of argon with cosmic ray protons and in soil and rock by neutron activation of potassium (K), calcium (Ca) and Cl (White and Broadley 2001). The resulting radiological dose to individuals can be estimated from the ratio of ^36^Cl to stable chlorine (^36^Cl/Cl) in the surface environment and varies with geographical location. The natural ^36^Cl/Cl ratio is between 10^−15^ and 10^−12^ (Campbell et al. 2003). The dose can thereby differ by several orders of magnitude between coastal and inland areas due to the difference in concentration of stable Cl. ^36^Cl/Cl ratios exceeding 10^−12^ (up to 2·× 10^−11^) were found in a 100-km^2^ area in the Tokaimura region, Japan, where four nuclear power reactors and one nuclear fuel reprocessing plant were once operated (Seki et al. 2007).

^36^Cl was produced in large amounts by neutron activation of seawater upon nuclear weapons testing between 1952 and 1958 (Peterson et al. 2007). These peaks in ^36^Cl have been used for dating ground water (Campbell et al. 2003; White and Broadley 2001). ^36^Cl is also produced during nuclear power reactor operation due to neutron capture of stable ^35^Cl that may be present at trace levels in core materials, graphite, coolant water and construction materials such as steel and concrete (Frechou and Degros 2005; Hou et al. 2007). In addition, ^36^Cl can be produced in considerable amounts via spallation reactions of other concrete components, such as K and Ca, primarily in fast reactors where high-energy particles such as fast neutrons are present (Aze et al. 2007). Although ambient ^36^Cl levels typically are low, the active uptake of Cl by organisms leads to higher concentrations in plants than in the soil in which they grow (Kashparov et al. 2007a, 2007b; White and Broadley 2001). Therefore, information about Cl cycling in soils, sediments and vegetation, including bioavailability and residence end exposure times, is necessary for relevant risk assessments (Limer et al. 2009).

## Major Cl reservoirs and large-scale cycling

The largest Cl reservoirs on the earth’s surface are the crust and the ocean (Graedel and Keene 1996) (Table 1). Inorganic Cl by far dominates these reservoirs. Estimates for the other reservoirs are also largely based on Cl^−^ concentration measurements. This assumption of a general dominance of Cl^−^ in the pedosphere is problematic as Cl_org_ have been shown to range from 11 to near 100% of the total Cl pool in a large range of soil types (Gustavsson et al. 2012; Johansson et al. 2003a; Redon et al. 2011; Redon et al. 2013); the pedosphere Cl pool may be at least twice as large if Cl_org_ is included.

**Table 1:** Major Cl reservoirs on earth, how they were estimated and theoretical residence times based on data from Graedel and Keene (1996) and updated with new data (in italic). The biosphere was not included in the former estimates of Cl reservoirs. Note that major pools of organic Cl are not considered and therefore the pedosphere reservoir is highly uncertain and may be at least twice as large (see text for details)

Reservoir	Cl content (g)	Reservoir was estimated from	Residence time (years)
Mantle	22 × 10^24^	Meteorite Cl:Si ratio; mantle mass	1.1 × 10^13^
Crust	60 × 10^21^	Meteorite Cl:Si ratio; crustal mass	3.4 × 10^8^
Oceans	26 × 10^21^	Cl concentration; water volume	4.3 × 10^6^
Freshwater	320 × 10^15^	Average Cl^−^ concentration in rivers and ground water; water volume	1.5 × 10^3^
*Pedosphere*	*48* × *10*^*15*^	*Average total soil Cl 200 μg g*^*−1*^ *d.m. (of which 50% is organically bound); mean soil depth (2 m) and a of density 1.0 g cm*^*−3a*^	5.3 × 10^2^
*Biosphere*	*0.9* × *10*^*15*^	*Estimated plant biomass (80% of total biomass) Cl*^*−*^ *concentration of 0.1 mg g*^*−1*^ *d.m.; global plant biomass 900 Gt*	
Cryosphere	0.5 × 10^15^	Cl^−^ concentration in rain or snow; ice volume	8.3 × 10^1^
Troposphere	5.3 × 10^12^	Concentrations of HCl, CH_3_Cl and Cl^−^aerosols; troposhere volume	8.8 × 10^−4^
Stratosphere	0.4 × 10^12^	Cl concentration; stratosphere volume	1.3 × 10^1^

^a^Assumptions by Graedel and Keene (1996); no published references in support of these values provided

Biosphere estimates of Cl pool have not been included in prior studies. If we assume a plant biomass concentration of Cl^−^ of 1 mg g^−1^ d.m. (dry matter), which the plant does not show deficiency symptoms (Marschner 2012) and a global plant biomass of 900 Gt (Bar-On et al. 2018), the plant biomass reservoir of Cl (0.9 × 10^15^ g) is considered smaller than the pedosphere Cl pool (48 × 10^15^ g).

Rapidly growing plants take up large amounts of Cl^−^. In common crops, the ratios of Cl^−^ concentrations in fresh plant tissue to Cl^−^ concentrations per dry mass in the top 20 cm of soil were 1.5–305 (Kashparov et al. 2007a, 2007b). This is in line with the high proportion, 10%, of total catchment Cl found in biomass from a Danish forest (Öberg et al. 1998). In coniferous forests, a much higher root uptake of Cl^−^ than the plant Cl^−^ demand was found resulting in large leaching and throughfall from the trees (Montelius et al. 2015; Redon et al. 2011; van den Hoof and Thiry 2012). The reasons for the excess uptake and the extensive internal ecosystem Cl^−^ cycling in some environments are presently unknown but can dramatically increase Cl^−^ residence times and soil concentrations (Montelius et al. 2015; Tanaka and Thiry 2020).

In the large-scale inorganic Cl cycle, mineral weathering contributes with Cl^−^ to freshwaters that subsequently reach the oceans (Graedel and Keene 1996). The largest contribution of Cl^−^ to the atmosphere is sea salt aerosols while minor contributions include HCl from volcanic activity and biomass burning, mineral aerosols and VOCls of natural or anthropogenic origin. Cl^−^ is transported to oceans and soils by wet and dry deposition. Fluxes between the reservoirs have been proposed (Graedel and Keene 1996), but these values are poorly constrained. For example, to balance the overall budget, a yearly Cl loss of 30 Tg year^−1^ from the pedosphere (equivalent to 1.25‰ of the total pedosphere reservoir) had to be assumed. Given the other budget elements, such a flux would lead to a rapid depletion of the pedosphere stock which is unrealistic. Such budget calculations illustrate the lack of relevant information regarding the large-scale fluxes and a possible bias caused by not including Cl_org_ in the budget.

## Cl in terrestrial ecosystems

During the past decades, there has been a rapid development towards improved qualitative understanding of the terrestrial Cl cycle. Below, we summarise ecosystem inputs, transformation, translocation and export processes, reservoirs and finally ecosystem budgets regarding Cl.

### Deposition

Prior to the mid-1950s, the chemical composition of surface waters was considered a result of physical land use in combination with the geochemical, hydrological processes and features of the surrounding area. In the mid-1950s, it was suggested that the chemical composition of rivers mirrors the chemical composition of precipitation (Eriksson 1955). The arguments were based on extensive data on Cl^−^ and sulphate in precipitation and rivers and implied that these ions originated from oceans (Eriksson 1960). The aerosols are carried with the winds and deposited either back to the sea or on land by precipitation that washes out Cl^−^ from the atmosphere. Gases and particles can also contain Cl^−^. These can undergo ‘dry deposition’, i.e. be deposited directly on the ground or vegetation, or ‘wet deposition’, i.e. washed out of the air by precipitation. The total deposition is generally higher in forested areas than over open land because atmospheric particles are intercepted by vegetation; leaching from the leaves is also possible.

The quantification of wet deposition of Cl^−^ can be done with high precision and is relatively well constrained but variable with higher depositions close to the sea and influenced by the prevailing winds (Clarke et al. 2009). For instance, in Europe, the average Cl wet deposition is approximately less than 20 kg ha^−1^ year^−1^ but varies from 0.5 to 220 kg ha^−1^ year^−1^ (Clarke et al. 2009). In contrast to wet deposition, dry deposition is difficult to measure as it, besides distance to the sea, also includes inputs via gases, aerosols and particles and is affected by interception by surfaces, e.g., tree canopies. Therefore, quantitative figures of dry deposition are considerably more uncertain than those of wet deposition but have been estimated to be 15–73% (average 43%) of total deposition based on data from North America and Europe (Svensson et al. 2012).

It is well known that precipitation, in addition to Cl^−^, also contains Cl_org_ (Enell and Wennberg 1991; Grimvall et al. 1991; Laniewski et al. 1995). Measurements of individual halogens in organic matter derived from precipitation show that most of the organically bound halogens detected as adsorbable organic halogens (AOX) are chlorinated compounds (Laniewski et al. 1999). Brominated compounds are widespread but less common, and organically bound iodine has only been detected at sites close to the sea (Laniewski et al. 1999).

A major part of Cl_org_ present in precipitation and snow consists of relatively polar semi- or non-volatile compounds, particularly organic bases and acids (Laniewski et al. 1999). Chloroacetic acids can occasionally explain up to 6% of the Cl_org_ in precipitation (von Sydow 1999) while the relative contribution from VOCls usually is smaller, often at ng L^−1^ concentrations (Schleyer 1996).

Little is known about the origin of the Cl_org_ in precipitation. Known industrial pollutants, such as flame retardants (e.g., chlorinated alkyl phosphates) and pesticides (e.g., lindane), are typically present at ng L^−1^ levels (Stringer and Johnston 2001), i.e. in concentrations about three orders of magnitude less than the total Cl_org_ concentrations. Chloroacetic acids (CCAs) have been detected in rain (Frank 1991; Reimann et al. 1996), but the origin is under debate (Berg et al. 2000; Cape et al. 2006; Laturnus et al. 2005). In addition, a study conducted at Klosterhede in northwest Denmark suggests that Cl_org_ in throughfall mainly originates from plant internal sources rather than from dry deposition (and thus external) sources (Öberg et al. 1998). Although there is important and extensive work on the formation and emissions of volatile organic halogens, their low concentrations in rain (ng L^−1^; Laniewski et al. 1999; Svensson et al. 2007a) cannot explain the total rain Cl_org_ levels (μg L^−1^; Öberg et al. 1998; Montelius et al. 2015).

### Weathering

As mentioned above, it has long been believed that Cl^−^ primarily participates in geochemical processes and is only negligibly affected by biological processes or interactions with organic matter. Riverine Cl^−^ has likewise often been considered to originate from the atmosphere only, despite possible weathering processes during the pathway through the soil (Eriksson 1960; Schlesinger and Bernhardt 2020). There are limited analyses of Cl^−^ in rocks, but felsic bedrocks such as granite contain low amounts of Cl^−^, and the highest amounts are found in mafic bedrocks (Melkerud et al. 1992) and, obviously, in halide-rich evaporites. Felsic minerals can be considered to have a lower chemical weathering rate than mafic minerals. The weathering rate has been estimated for a small stream at Hubbard Brook, New Hampshire, USA, with bedrock consisting mainly of granite, and approximately 4–8% of the Cl^−^ stream output originated from weathering (Lovett et al. 2005).

Land rise in previously glaciated regions can result in soils originating from marine sediments and therefore are rich in Cl^−^. Release of Cl^−^ from such marine deposits constitutes a special case with potentially significant subsurface contribution of Cl^−^ to soils, water and organisms.

### Input from irrigation, fertilisation and road de-icing

Anthropogenic Cl^−^ input from irrigation and fertilisation can represent substantial inputs to terrestrial environments. Irrigation with low to medium level salinity water can contribute in the order of 500–1000 kg ha^−1^ year^−1^, i.e. anthropogenic contributions can be the major Cl^−^ input (Xu et al. 2000).

Since the start of de-icing of roads in mid-twentieth century, studies have shown increased Cl^−^ concentrations in both surface water and groundwater in the vicinity of roads (Dugan et al. 2017; Kaushal et al. 2005; Kelly et al. 2008). Road salt effects can be chemical, e.g. induce ion exchange affecting acidification and metal and nutrient leaching (Bäckström et al. 2004; Löfgren 2001), or biological, i.e. affect aquatic food webs (Hintz and Relyea 2019; Todd and Kaltenecker 2012; Van Meter et al. 2011). The application of road salt has been estimated to be substantial compared with deposition (Forczek et al. 2011), and there are indications of associated extra Cl retention in soil (Robinson et al. 2017; Kincaid and Findlay 2009; Perera and Gharabaghi 2013).

### Processes driving terrestrial Cl cycling

Most of the soil chlorination is driven by enzymes, i.e. organisms, but abiotic chlorination seems also to occur at significant rates (Bastviken et al. 2009; Rohlenova et al. 2009; Matucha et al. 2006; Weigold et al. 2016; Atashgahi et al. 2018). Chlorination rates have been experimentally investigated at temperatures ranging from 4 to 50 °C, where low rates were found at 4 and 50 °C, possibly representing primarily abiotic rates near the temperature extremes, while there was an enzymatic response pattern with a maximum at 20 °C indicating a dominance of biotic chlorination at many common temperatures. There are indications that abiotic processes related to iron cycling in soils contribute to Cl^−^ retention (Fahimi et al. 2003; Keppler et al. 2000). However, since the redox cycling of iron is usually a consequence of microbial activity, the proposed abiotic processes may be linked to biological processes, albeit indirectly. Under experimental conditions, formation of volatile Cl_org_ in hypersaline lake sediments from Western Australia is higher under biotic conditions than in sterilised samples but were not stimulated via Fe redox transformations or the formation of reactive Fe species (Ruecker et al 2014).

Chlorination of organic matter can occur both inside and outside cells. The intracellular chlorination seems strictly regulated by enzymatic processes (van Pee and Unversucht 2003). Enzymes known to mediate intracellular chlorination include FADH_2_-dependent halogenases and perhydrolases. In addition, biotic chlorination has been suggested to involve an enzymatic reaction, where methyl halide transferase catalyses the formation via the reaction of S-adenosyl-l-methionine (SAM) with chloride (Wuosmaa and Hager 1990; Atashgahi et al. 2018). The underlying process for the extracellular chlorination seems to be a formation of reactive chlorine (e.g. hypochlorous acid, HOCl) formed by reactions between hydrogen peroxide and Cl^−^ (Neidleman and Geigert 1986). Reactive Cl is a strong oxidant and reacts with surrounding organic matter rendering unspecific chlorination of various organic compounds in the large and complex pool of soil organic matter (Hoekstra et al. 1998a; van Pee and Unversucht 2003). Extracellular chlorination also depends on the production of reactive chlorine by enzymes, such as heme and vanadium containing haloperoxidases, but the enzymatic control is less rigorous compared with intracellular chlorination (van Pee 2001).

Given the rapid chlorination rates and consequent Cl^−^ retention in soil, ubiquity of Cl_org_ and the widespread capacity among organisms to perform/promote chlorination, a fundamental, albeit unknown, ecological/evolutionary explanation for organic matter chlorination is likely. Intracellular chlorination processes have been explained as ways of detoxification or are believed to be produced as chemical defence compounds (e.g. antibiotics), hormones or pheromones (Hoekstra et al. 1998b). However, direct verification of these hypotheses is limited. Extracellular chlorination represents a different process, although it is well documented that reactive chlorine species such as hypochlorous acid are potent bactericides used by phagocytes to kill invading microorganisms (Apel and Hirt 2004). Several microorganisms and plants produce chlorinated allomones, i.e. substances that deter or kill competing or pathogenic organisms. The ability to use reactive chlorine in the chemical defence against competing microorganisms could provide a substantial advantage and be evolutionarily favoured. Indeed, screening genetic databases for identified haloperoxidases from terrestrial environments indicates that many originate from organisms associated with plants or decomposing plant material; the ability to produce reactive chlorine may be especially common in environments that are known for antibiotic-mediated competition for resources (Bengtson et al. 2009).

Another hypothesis relates to microbial processing of organic material representing their substrates. There is a general perception that chlorinated organic matter is less bioavailable than non-chlorinated organic compounds. However, chloroperoxidases, like many other oxidases, catalyse production of small reactive molecules (hypochlorous acid in the case of chloroperoxidase) that can break C–C bonds in complex, refractory organic compounds (Hoekstra et al. 1998b; van Pee and Unversucht 2003) whereby smaller, more bioavailable fragments of the refractory compounds may be formed. The exposure of lignin to reactive chlorine enhances its biodegradability, (Johansson et al. 2000), and fungal chloroperoxidase activity results in depolymerisation and breakdown of synthetic lignin supports this hypothesis (Ortiz-Bermúdez et al. 2003). Similarly, biodegradation of lignin in the effluent of chlorine bleached pulp mills is higher than the degradation of corresponding chlorine-free lignin (Bergbauer and Eggert 1994). Hence, promoting formation of Cl_org_ could be a way of increasing the organic substrate supply for microorganisms as these compounds could be preferred as substrates by microorganisms after dechlorination.

A third potential reason for microbial chlorination is defence against oxygen radicals. Formation of reactive chlorine is related to consumption and detoxification of reactive oxygen species including hydrogen peroxide and oxygen radicals; by formation of, e.g., extracellular hypochlorous acid, reactive oxygen species may be prevented from entering the cell. Interestingly, repeated oxidative stress exposure induces the expression of chloroperoxidase genes and increases the production of reactive chlorine in some algae and bacteria (Bengtson et al. 2013).

Establishing how various environmental factors regulate chlorination and influence chlorination rates is important for understanding Cl cycling. Several hypotheses have been proposed, but these need further clarification. Tests with different nitrogen levels are ambiguous and local variability large (Bastviken et al. 2006; Rodstedth et al. 2003). Chlorination rates are slower under anoxic conditions than oxic (Bastviken et al. 2009), which is reasonable given that chlorination of organic matter is an oxidative process. This indicates an indirect regulation by soil moisture, but the overall regulation of natural chlorination of organic matter is still unclear. Varying the nitrogen levels has yielded ambiguous results (Bastviken et al. 2006, Rodhstedt 2000), and local variability seems large. A factorial study showed that total chlorination was hampered by addition of nitrate or by nitrate in combination with water but enhanced by addition of chloride as well as labile organic matter (glucose and maltose) (Svensson et al. 2017). These estimates were based on studies of bulk soil excluding roots, which likely has underestimated the chlorination potential as it was later suggested that most of the chlorination takes place in the rhizosphere (Montelius et al. 2019).

Dechlorination processes (transformation from Cl_org_ to Cl^−^ by either organic matter decomposition or by selective removal of Cl atoms from organic molecules) have been extensively studied in relation to Cl_org_ pollution and bioremediation (van Pee and Unversucht 2003). Chlorinated compounds can be used as terminal electron acceptors in microbial metabolism. Interestingly, the Gibbs-free energy yield of this process is similar to the energy yield with nitrate as the electron acceptor and only slightly lower than the energy yield of oxic respiration (Smidt and de Vos 2004). Hence, chlorinated organic compounds can be very potent as electron acceptors. Dechlorination could therefore be the result of either degradation of the chlorinated organic matter or the microbial use of chlorinated organic molecules as electron acceptors, i.e. organohalide respiration; there is a wide literature regarding dehalogenation processes in terms of biochemistry of specific compounds (Dolfing 2000; Fetzner 1998; Olivas et al. 2002; Pries et al. 1994; Smidt and de Vos 2004; van Pee and Unversucht 2003). There are also studies that show net-uptake of chloromethane without evidence of organohalide respiration (Peng et al. 2020), and a cometabolic dechlorination has been suggested (Atashgahi et al. 2018; Peng et al. 2020). However, rates and regulation of dechlorination of bulk Cl_org_ in nature are still rarely quantified although the limited available information suggests rates are high (Montelius et al. 2016). Studies have shown a pH dependence of inhibition of dechlorination and alternative electron acceptors (Paul and Smolders 2014; Aulenta et al. 2007; Yang et al. 2017). Further studies on the activities are needed to confirm dechlorination in non-contaminated soils, as organohalide respiring organisms, using Cl_org_ as electron acceptors for growth, are likely present and active in different types of uncontaminated soils (Krzmarzick et al 2011; Montelius et al. 2016; Zlamal et al. 2017). There are already results on that the potential of dechlorination in terrestrial environments is both common and widespread with a variety of genes encoding for enzymes capable of dehalogenation (Temme et al. 2019).

Directly measured dechlorination rates of bulk Cl_org_ in terrestrial environments indicate that soil Cl_org_ levels result from a dynamic equilibrium between the chlorination and rapid dechlorination of some Cl_org_ compounds, whereas another Cl_org_ pool is dechlorinated more slowly (Montelius et al. 2016). Hence both chlorination and dechlorination processes are important for understanding the behaviour of Cl^−^ in soils, and it has even been suggested that the balance between chlorination and dechlorination is more important for soil Cl^−^ levels than Cl^−^ deposition (Gustavsson et al. 2012; Montelius et al. 2015).

Soil microorganisms seem capable of rapidly taking up large quantities of Cl^−^ during growth phase adding to ecosystem Cl dynamics (Bastviken et al. 2007). After addition of ^36^Cl to experiment soil, 20% was incorporated into microbial biomass within 5 days (Bastviken et al. 2007), suggesting that rapid microbial growth following a system disturbance (a rain event, leaf fall in autumn etc.) could lead to rapid microbial uptake of Cl^−^ based on physiological need. It is unclear if this can affect Cl_org_ formation rates.

## Terrestrial reservoirs of chlorine

### Soil

Total Cl typically range from 20 to > 1000 mg kg^−1^ d.m. in non-saline soils (Table 2). The concentrations of Cl_org_ in surface soil are in most cases higher than Cl^−^ concentrations (Table 2). The dry mass fraction of Cl_org_ in surface soils (0.01–0.5%) is in parity with that of phosphorous (0.03–0.2%) and only slightly lower than nitrogen (1–5%) and sulphur (0.1–1.5%). Bulk density, horizon thicknesses and stoniness affect total storage of Cl more than concentration differences. Unfortunately, these parameters make the calculation of total Cl storage capacity difficult. Total storage is usually largest in the mineral soil layer because of its greater thickness compared with the organic surface layer, although Cl_org_ concentrations are typically 2–5 times higher in the organic surface soil layer (Redon et al. 2011). Available data only separates organic and mineral soil layers, and more detailed discussions about specific soil profiles are therefore not possible at this point. Cl_org_ levels are higher in soils with more organic matter (Redon et al. 2011), while the percentage Cl_org_ is frequently higher (> 80%) in mineral soils (Table 2). Hence, higher soil total Cl levels may relate to soil organic matter content, probably because soil chlorination processes retain and accumulate Cl as Cl_org_ (Gustavsson et al. 2012, Montelius et al. 2015). However, available information is scarce and non-conclusive, but high levels of Cl_org_ (average 245 μg g^−1^) have been found in coastal Arctic wet tundra soils (Zlamal et al. 2017).

**Table 2:** Total Cl concentrations and the fraction Cl_org_ in various soils. Soil depth is denoted by soil layer (e.g. humus and mineral layers) or by distance from soil surface

	Ecosystem, country	Total Cl (mg kg^−1^)	Cl_org_ (%)	Soil layer/depth	Source
Litter	Coniferous forest and deciduous forest France	45–528	11–100	Litter	Redon et al. (2011)
Humus	Con. forest, Sweden	99–274	67–73	Humus	Gustavsson et al. (2012)
	Conif. forest, Sweden	154	86	Humus	Bastviken et al. (2009)
	Conif. forest, Sweden	331	95	Humus	Bastviken et al. (2007)
	Conif. forest, Sweden	127	69	Humus+ mineral	Svensson et al. (2007)
	Conif. forest, Sweden			Humus	Öberg et al. (2005)
	Conif. forest, Sweden	369–458	81–85	Humus	Johansson et al. (2003, Johansson et al. (2003)
	Conif. forest, Sweden	310	68	Humus	Johansson et al. (2001)
	Conif. forest, Denmark	206–772	67–85	Humus	Albers et al. (2010)
	Conif. forest, China	45	38	15 cm	Johansson et al. (2004)
	Conif. and decid. forests, France^a^	45–1041	40–100	Humus	Redon et al. (2011)
	Mixed decid. forest, Sweden	224	85	Humus	Johansson et al. (2003), Johansson et al. (2003)
	Conif. forest Sweden	277–353	72	FH	Svensson et al. (2013)
Mineral	Mixed forests, France^a^	34–340	89	Mineral, 0–30 cm	Redon et al. (2013)
	Conif. and decid. forests, France^a^	25–210	29–100	Mineral 0–10 cm	(Redon et al. (2011)
	Conif. and decid. forests, France^b^	31–35	60–78	30–45 cm	Montelius et al. (2015)
	Conif. and decid. forests, France^b^	25–29	57–84	45–60 cm	Montelius et al. (2015)
	Conif. and decid. forests, France^b^	23–32	50–71	60–75 cm	Montelius et al. (2015)
	Conif. forest, Sweden	16–27	100	E	Svensson et al. (2013)
	Conif. forest, Sweden	65–102	69	Bs	Svensson et al. (2013)
Other soils	Pasture, Sweden	46–65	85–90	5–15 cm	Gustavsson et al. (2012)
	Grassland, France^a^	13–1248	83	Mineral, 0–30 cm	Redon et al. (2013)
	Agricultural soil, France^a^	19–100	87	Mineral, 0–30 cm	Redon et al. (2013)
	Agricultural soil, Sweden	45–49	84–89	5–15 cm	Gustavsson et al. (2012)
	Paddy soil, China	38	34	15 cm	Johansson et al. (2004)
	Peat bog, Canada	30–1177	43–84	Surface—6 m	Silk et al. (1997)
	Peat bog, Chile	366–1084	82–93	Surface—2 m	Biester et al. (2004)
	Sub-arctic/arctic soil, Greenland and Sweden	84–242	42–97	10 cm	Albers et al. (2017)

^a^Includes study sites at different distances to the sea

^b^Includes sites studied with different tree species

Despite the large number of identified chlorinated organic compounds, the molecular composition of the bulk Cl_org_ in soils is largely unknown. The soil organic matter seems mainly composed of high molecular mass substances, usually > 1000 Dalton (Hjelm and Asplund 1995). However, Cl_org_ content in different types of soil organic matter has rarely been determined. Of the Cl_org_ in coniferous soil, 1–10% was associated with water-leachable fractions of the organic matter (Bastviken et al. 2009, 2007). Further, in lysimeter soil, Cl_org_ is associated with organic matter with a molecular mass < 10 000 Dalton, while most organic matter had molecular mass, i.e., > 10,000 Dalton (Lee et al. 2001).

The concentration of Cl_org_ is usually found higher in coniferous forest than deciduous forest soils (Johansson et al. 2003a). This pattern was confirmed by Redon et al. (2011) in a study of more than 50 forested sites in France. In addition, soil humus in plots with Norway spruce (*Picea abies*) had higher net accumulation of Cl^−^ (7 times) and Cl_org_ (9 times) than soil humus of plots with Sessile oak (*Quercus sessiliflora*) over an experimental period of 30 years (Montelius et al. 2015). Thus, it seems that vegetation characteristics can explain local soil Cl^−^ and Cl_org_ levels, which may explain why these are independent of atmospheric deposition.

### Water

In contrast to soils, Cl^−^ concentrations generally exceed Cl_org_ concentrations in water. For example, the Cl^−^ concentration in various waters is measured in mg L^−1^, while Cl_org_ is typically measured in μg L^−1^ and VOCls are in the range of ng L^−1^ (Table 3) (Asplund and Grimvall 1991; Enell and Wennberg 1991; Eriksson 1960, McCulloch 2003). Hence, the atmospheric deposition of Cl_org_ is three orders of magnitude lower than deposition of Cl^−^ and thereby often assumed to be negligible from a total Cl perspective. While ground water has higher Cl^−^ concentrations than precipitation and surface waters, Cl_org_ and VOCl concentrations can be higher in surface waters than in precipitation (Table 3).

**Table 3:** Chloride (Cl^−^), organochlorines (Cl_org_) and chloroform concentrations in various waters, primarily in Sweden. Chloroform is one of the most frequently detected volatile chlorinated organic compounds (VOCl) in surface water

	Cl^−^ (mg L^−1^)	Cl_org_ (μg L^−1^)	Chloroform (ng L^−1^)
Precipitation	0.2–3.5^a^	1–15^d^	11–97^g^
Groundwater	10–300^b^	5–24^e^	5–1600^h^
Surface water (lakes and rivers)	0.74–11^c^	5–200^f^	4–3800^i^

^a^Minimum and maximum concentrations obtained from 6 precipitation stations in different regions of Sweden 1983–1998 (Kindbohm et al. 2001)

^b^Minimum and maximum concentrations from 20,100 wells (dug wells and drill wells) in Sweden sampled during 1984–1986 (Bertills 1995)

^c^Concentrations (10th and 90th percentiles) obtained from analyses of Swedish lakes during 1983-1994 (Wilander 1997)

^d^Minimum and maximum concentrations in precipitation and snow at 7 sites in Sweden (Laniewski et al. 1999; Laniewski et al. 1995), combined with typical range given in Öberg et al. 1998 (Öberg et al. 1998)

^e^Minimum and maximum concentrations in groundwater from 14 wells in Denmark (Grön 1995)

^f^Minimum and maximum concentrations in 135 lakes (Asplund and Grimvall 1991) and rivers in Sweden (Enell and Wennberg 1991)

^g^Minimum and maximum concentrations of chloroform obtained from precipitation measurements in Germany 1988–1989 (Schleyer 1996; Schleyer et al. 1991)

^h^Minimum and maximum concentrations obtained from groundwater measurements at one site in Denmark (Laturnus et al. 2000)

^i^Minimum and maximum concentrations compiled from rivers and lakes in Belgium, Canada, France, Germany, The Netherlands, Switzerland, UK, USA (McCulloch 2003)

### Sediment

Analysis of sediment Cl_org_ has usually focused on contamination from industrial activities (Jonsson 1992; Poykio et al. 2008). There is a large body of literature on specific chlorinated pollutants (e.g. polychlorinated biphenyls [PCB] and dichlorodiphenyltrichloroethane and its degradation products [DDTs]). Among the bulk Cl_org_ measurements, AOX has been used to study sediment pore waters, but such efforts in non-contaminated sediments are rare. The AOX method has been adapted for other types of bulk Cl_org_ analyses including extractable organic halogens (EOX) and volatile organic halogens (VOX). There has also been a suggestion to avoid AOX analyses for sediment pore waters as it does not discriminate between natural and anthropogenic Cl_org_ (Müller 2003). Extractable organic halogens (EOX; extraction of sediments with cyclohexane–isopropanol under sonication) yielded concentrations of 5–70 μg g^−1^ (lipid mass) in the upper 2 cm of Bay of Bothnia sediments in an area with contamination from pulp and paper mills to evaluate the effects of chlorine bleaching (Poykio et al. 2008). Another study reported Cl concentrations of < 10 to 840 μg g^−1^ organic matter in seven non-polluted inland water sediments (Suominen et al. 1997). The analysis methods differed (AOX and EOX after various extractions) making comparisons difficult. Analyses using similar methodology as for soils, such as total organic halogens (TOX), appear to be largely missing, and therefore, Cl and Cl_org_ levels in sediments are presently unclear.

### Biomass

The Cl^−^ content of plant biomass varies between species. For plant growth, a general Cl^−^ requirement of 1 mg g^−1^ d.m. has been suggested; deficiency symptoms have been observed at 0.1–5.7 mg g^−1^ d.m., while toxicity has been reported at 4–50 mg g^−1^ d.m. (White and Broadley 2001). Thus, extrapolations across species are uncertain. Plant Cl_org_ content has been estimated to 0.01–0.1 mg g^−1^ d.m. (Öberg et al. 2005), but this is based on scattered measurements from beech (*Fagus sylvatica*) leaves, spruce needles, *Sphagnum* moss and bulk samples of grass, and the variability between species and plant parts is unknown at present. Table 4 shows a large variation in different types of vegetation.

**Table 4:** Reported average (min-max) concentrations of Cl in different types of vegetation. (*DM* dry matter)

	Vegetation	TotCl (μg g^−1^ DM)	Cl^−^ (μg g^−1^ DM)	Cl_org_ (μg g^−1^ DM)	Reference
Tree layer	Foliage	1214 (183–14,264)	266 (110–590)	37 (10–150)	*a–f*
	Wood	80 (6–530)	74 (9–380)	26 (3–150)	*b,d,e*
	Branches	66 (26–133)	55 (13–121)	8 (6–12)	*d,e*
	Bark	98 (26–296)	87 (19–281)	9 (2–15)	*d,e*
Field layer	Aboveground plant	1237 (116–4420)	1131 (28–4241)	107 (10–243)	*b,g,h,i*
Bottom layer		757 (541–1030)	575 (285–800)	154 (60–265)	*b,h,i*

^a^Holmes and Baker (1966)

^b^Flodin et al. (1997)

^c^Lovett et al. (2005)

^d^Montelius et al. (2015)

^e^Gielen et al. (2016)

^f^Edwards et al. (1981)

^g^Asplund and Grimvall (1991)

^h^Nkusi and Müller (1995)

^i^Zlamal et al. (2017)

Based on measurements of different plant parts, the standing stock Cl in trees in a pine forest in Belgium was estimated to be 4.7 and 5.5 kg ha^−1^ for wood plus leaves, and roots, respectively (van den Hoof and Thiry 2012). Fresh leaves had the highest Cl concentration (0.59 mg g^−1^ d.m.) corresponding to 35% of the total Cl in the trees. Cl_org_ accounted for less than 10% of the Cl in the leaves and the bark but constituted 20% of the total biomass Cl of the whole tree. Cl is an essential element, but the potentially high enrichment levels indicate that the role of Cl for trees is not fully understood, and that processes regulating Cl uptake in vegetation affect the residence times of chlorine in terrestrial ecosystems (Epp et al. 2020; Tanaka and Thiry 2020).

Investigation of total Cl in various landscape compartments, including soil, sediment, water and biomass, indicates that the terrestrial biomass Cl pool dominates over other pools and account for in the order of 60% of the total catchment Cl (Tröjbom and Grolander 2010). Cl was substantially enriched in biomass compared with other comparable elements (e.g., bromine and iodine) and nutrients (nitrogen, phosphorus, potassium, calcium). Cl is an essential element, but this level of enrichment indicates that the roles of Cl for organisms may not be fully understood, and that a large part of potential contaminant ^36^Cl reaching terrestrial parts of the landscape will be taken up by biota.

In terrestrial vascular plants alone, a couple of hundred chlorinated compounds have been identified (Gribble 2010), but many of these are relatively short lived and have a specific function, e.g. as auxins (Engvild 1994; Walter et al. 2020). Of interest for risk assessment are compounds that are toxic, and which may be transferred between organisms in the food chain. Several natural chlorinated compounds have structural similarities with persistent organic pollutants (POPs) that are believed to be produced at low trophic levels in algae and sponges (Gribble 2010). Many clearly accumulate through the food chain; very high concentrations may occur in, e.g. cetaceans (Alonso et al. 2012; Mwevura et al. 2010).

Several types of natural halogenated carboxylic acids are known (Dembitsky and Srebnik 2002). Most of these are structurally complex, often containing reactive functional groups that shorten their environmental half-lives. However, chlorinated fatty acids (ClFAs) have both a long half-life in the environment and undergo food-chain transfer (Björn 1999; Mu et al. 2004). ClFAs constituted the bulk of extractable organically bound chlorine (EOCl; 70% or more) in environmental samples in the 1990s (Håkansson et al. 1991; Mu et al. 2004). To date, the origin of ClFAs is not understood. There are clearly anthropogenic influences as EOCl concentrations are particularly high close to point sources of Cl_org_. An important type of point source was production of chlorine-bleached pulp and paper (Håkansson et al. 1991), but there are also reports indicating that the ClFA-profile depends on the type of anthropogenic pollutants present (Vereskuns 1999). Research is lacking, but as far as known, ClFAs are ubiquitous and present also in areas where the concentrations of anthropogenic POPs are low. Thus, *de novo* synthesis of ClFAs in organisms is possible, and formation of CLFAs via chloroperoxidase action has been shown in some marine organisms (Mu et al. 2004).

ClFAs are particularly interesting from a risk assessment point of view as they are not recognised by biota as xenobiotics although they may exert toxicity (Ewald 1998). In contrast to legacy POPs, ClFAs do not primarily accumulate in fatty tissues but are incorporated in membrane lipids; the concentrations of ClFAs relative other fatty acids (FAs) are higher in muscle tissues than in depot fat, at least in mammals (Björn 1999; Åkesson Nilsson 2004). Of particular interest for this review, a *de novo* synthesis of ClFAs incorporating ^36^Cl released into the environment could potentially introduce a long-lived source of radioactivity in areas where critical membrane functions take place.

A possible complication in assessing the environmental problems is that toxicity, detoxification pathways and uptake or emission of halogenated compound in terrestrial or aquatic environments may change upon dehalogenation (Skladanka et al. 2012). For full understanding of possible ecotoxic problems, the toxicity of individual compounds as well as their degradation products may have to be considered (Weissflog et al. 2007). Some compounds may also react directly with compounds that are key for cell function. For example, as halogens are leaving groups, halogenated aliphatics my react with endogenous or exogenous nucleophiles, including DNA and proteins (Motwani et al. 2011), affecting the efficiency of biological processes.

### Litter

Simultaneous leaching of Cl^−^ and formation of Cl_org_ has been shown in litter (detached dead or dying plant biomass) (Myneni 2002). A study of senescent leaves from white oak (*Quercus alba*) showed Cl^−^ and Cl_org_ contents of 335 and 165 mg kg^−1^ (Leri and Myneni 2010). Cl fractions were quantified using X-ray absorption near-edge structure (XANES) spectroscopy. The results were that (1) total Cl_org_ content in the leaves increased during the senescence and gradual degradation of the organic matter, (2) aliphatic Cl_org_ was present at stable levels over time and seems contributed by plant processes and resistant to degradation and (3) that water-soluble aromatic Cl_org_ was first leached from the leaves followed by later accumulation of non-soluble aromatic Cl_org_ during senescence. There are however few studies that have quantified chlorine content in litter. Cl_org_ in spruce needle litter in Denmark was 51–196 mg kg^−1^ d.m. (median 101) (Öberg et al. 1998). In a study of 51 different forest sites in France with both coniferous and broad leaf tree species, total Cl content in the litter was 46–528 mg kg^−1^ d.m. (median 147), and the percentage Cl_org_ was 11–100% (median 40%) (Redon et al. 2011). Again, available data suggest substantial variability within and between species and locations.

## Translocation within systems

There are scattered indications of extensive internal cycling of Cl in terrestrial ecosystems. For example, the annual root uptake of Cl by Scots pine (*Pinus silvestris*) was nine times larger than the Cl demand by the tree (van den Hoof and Thiry 2012). The excess Cl was returned to soil primarily as Cl^−^ in throughfall Cl deposition and to some extent by litterfall. Similarly, a study integrating data from 27 forests of different types in France show that throughfall was variable, but on an average twice as high as the total atmospheric Cl deposition, 41 and 20 kg ha^−1^ year^−1^, respectively, while the average Cl in litterfall ranged from 0.1 to 2.5 kg ha^−1^ year^−1^ (Redon et al. 2011). This cycling within trees and soil, if a general phenomenon, will prolong Cl residence times in the forest ecosystem. The reasons for excess uptake of Cl relative to needs are unknown but could be related with evapotranspiration if Cl^−^ enters the plant with the water without discrimination. Therefore, vegetation seems to have a prominent role in the production and distribution of organohalogens.

The plant root-soil interface, the rhizosphere, is dynamic with numerous biogeochemical processes taking place that are important for terrestrial carbon cycling and other element cycling that sustain plant growth. Through root exudation of various chemical compounds, roots may regulate the nearby soil microbial community, plant defence, attracting beneficial microbes, change the chemical and physical properties of the soil or inhibit the growth of competing plant species (Philippot et al. 2013). The majority of the studies on Cl biogeochemical cycling have hitherto focused on bulk soil in which plant roots were removed by sieving, despite the knowledge that vegetation and vegetation-associated organisms have a strong influence on element turnover (Clemmensen et al. 2013; Pausch and Kuzyakov 2018). In a recent experimental study, we observed that increased availability of labile organic matter increased the Cl_org_ formation rates (Svensson et al. 2017). Hence, it seems that the most labile organic matter was rapidly degraded and fuelled a greater chlorination rate before being depleted. This indicates that increased chlorination rates are to be expected whenever more labile organic matter is present, e.g. in zones with more root exudate. These findings agree with the idea that chlorination is driven by biotic activity, which is highest in surface soil layers with higher root density and more input of organic material. Hence, root exudates in combination with plant-specific microbial interactions in the root zone may be important. Recent results from a radiotracer (^36^Cl) soil-plant (wheat, *Triticum vulgare*), the experiment shows that most halogenation took place in the rhizosphere. The specific halogenation rates (day^−1^) in soil with plants was at least two orders of magnitude higher (0.01 d^−1^) than without plants (0.0007 day^−1^), suggesting that plants play an active role in halogenation processes (Montelius et al. 2019).

There is also an extensive cycling of Cl in soils. Microbial activity in the topsoil results in formation of Cl_org_ from Cl^−^ (Bastviken et al. 2009; Öberg et al. 2005). In incubated soil samples from 14 sites, the amount of Cl converted to Cl_org_ in soil dry matter via microbial chlorination was 1.4–90 ng g^−1^ day^−1^ or 0.2–3‰ of the Cl^−^ pool being transformed to Cl_org_ per day (Gustavsson et al. 2012). Estimates based on field data are rare, but one study estimated the net chlorination to be 2 kg ha^−1^ year^−1^ from mass balance calculations, while laboratory estimates using the same soil yielded gross chlorination rates of 2–13 kg ha^−1^ year^−1^ corresponding to 50–300% of the wet deposition to this catchment (Bastviken et al. 2009; Bastviken et al. 2007). This discovery challenges the use of chloride as a tracer of water movement in the soil, in turn undermining many current hydrological and contaminant transport models. Substantial chlorination of organic matter occurs in agricultural, forest and peat soils (Gustavsson et al. 2012; Redon et al. 2013; Silk et al. 1997), implying that natural chlorination is a general and widespread phenomenon across many biomes and not limited to certain types of environments. The extensive natural chlorination of organic matter in soil suggests that the Cl turnover likely is linked to common ecosystem processes. Indeed, the chlorination rates were linked with microbial activity (Svensson et al. 2017), but the fundamental reasons for the extensive soil Cl-cycling are still unclear.

Available data (Table 5) indicates higher chlorination rates on a mass basis in litter compared with deeper soil layers. Extensive chlorination has been shown upon litter degradation (Myneni 2002), and seasonal patterns, with an increased aromatic Cl_org_ concentration during summer months, have been suggested (Leri and Myneni 2010).

**Table 5:** Examples of estimated soil organic matter chlorination rates. Note that all lab studies are conducted on bulk soil without rhizosphere except in the study by Montelius et al. (2019)

Type of study and experiment time	Specific chlorination(day^−1^)	Mass-based rate(ng Cl_org_ g^−1^ C_org_ day^−1^)	Area-based rate(kg Cl ha^−^1 year^−1)^	Source
Lab: arable soil; 11 weeks	0.00199	–	–	Lee et al. (2001)
Lab: agricultural soil: 4 months	0.00032–0.00055	2.6–5.0	–	Gustavsson et al. (2012)
Lab: peat; 8 weeks	0.00066	–	–	Silk et al. (1997)
Lab: forest soil; 78 days	0.00029	20	–	Bastviken et al. (2007)
Lab: forest soil; 6 months	0.0007–0.0034	78–311	–	Bastviken et al. (2009)
Lab: forest soil 25 days Without plant With plant	0.0007 0.001	5.6 90		Montelius et al. (2019)
Lab: conif. forest soil: 4 months	0.00094–0.0014	37–90	–	Gustavsson et al. 2012)
Lab: pasture soil: 4 months	0.00021–0.00074	3.5–4.9	–	Gustavsson et al. (2012)
Field experiment: spruce litter	–	1002	0.5	Öberg et al. (1996)
Field study: spruce litter	–	1469–7517	0.35	Öberg et al. (1998)
Lysimeter: mass balance			2.7	Öberg et al. (2005)
Catchment: mass balance			2.0	Öberg et al. (2005)

**Fig. 1:**
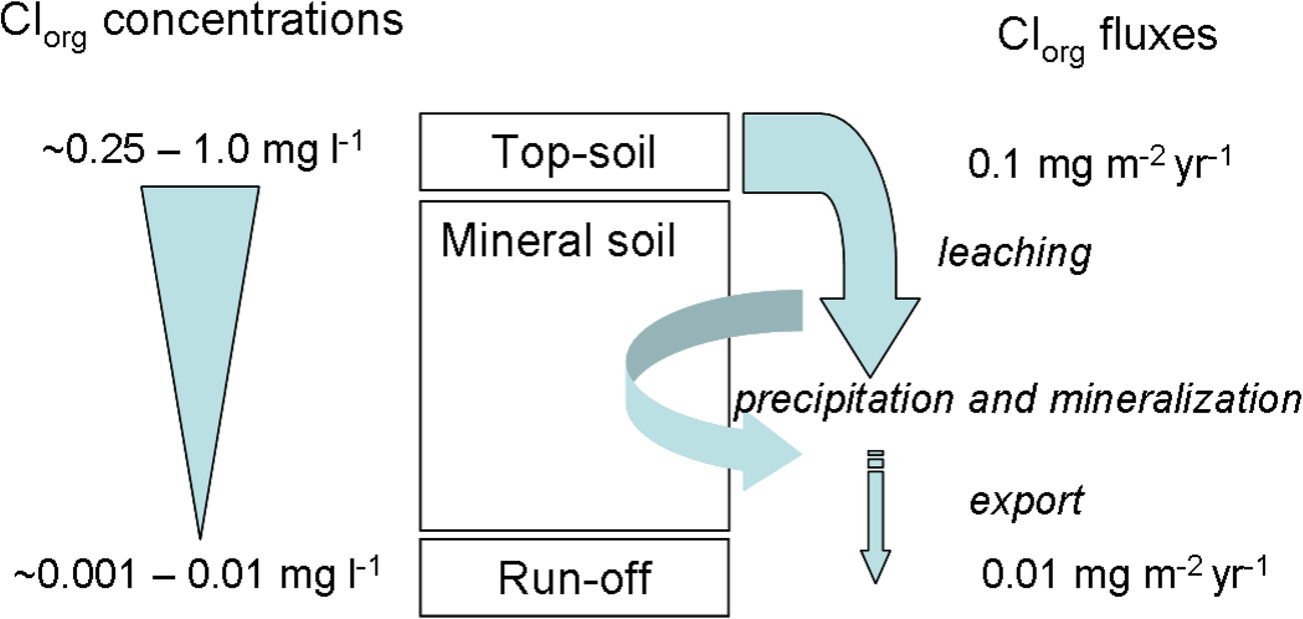
Estimated organic chlorine transport in forest ecosystem soil. Concentration data and flux estimations for topsoil are based on data from Rodhstedt (2000) and Svensson et al. (2007) leached from the topsoil and gradually lost from the soil water by precipitation or adsorption to the solid phase or by organic matter degradation while the water moves downward through the soil column. Cl^−^ shows an opposite pattern with lower relative concentrations in surface soils and increasing concentrations downward partly due to the release of Cl^−^ from Cl_org_. This model can explain why the water released from soils has higher concentrations of Cl^−^ than Cl_org_, while Cl_org_ dominates in surface soils

Migration through the profile may be important for internal Cl cycling in soils. Intensive chlorination has been observed in surface soil and litter layers, while Cl_org_ levels decrease with soil depth, and the form of Cl dominating in the hydrological export from catchments is Cl^−^ suggesting that Cl_org_ is leached from surface upland soils and either retained and preserved or transformed to Cl^−^ in deeper soil layers (Fig. 1) (Rodstedth et al. 2003; Svensson et al. 2007b; Öberg et al. 2005). Wetland soils also appear to retain Cl^−^, as a significant retention of road salt, 4–41%, has been indicated (McGuire and Judd 2020).

VOCls are produced in a wide variety of ecosystems such as wetlands, salt marshes and forests (Albers et al. 2010; Dimmer et al. 2001; Rhew et al. 2002), and in different climatic regions including arctic, temperate and tropical areas (Albers et al. 2017; Rhew et al. 2008; Jiao and Ruecker 2018; Yokouchi et al. 2002). Despite the growing knowledge of the field, data on VOCl emission rates are scattered and inconsistent. Budget and transport estimates on various scales are uncertain, partly because low concentrations of each specific VOCl make sampling and analyses challenging (Pickering et al. 2013). Furthermore, VOCl sources and sinks in terrestrial environments are not well understood, and continuous observations over time are scarce.

VOCls have been found in several terrestrial biomes such as tropical forest, grasslands, deciduous angiosperm forests, taiga, tundra and rice fields (Dimmer et al. 2001; Frank et al. 1989; Haselmann et al. 2002; Hoekstra et al. 2001; Khalil et al. 1998; Laturnus et al. 2000; Redeker et al. 2000; Rhew et al. 2008; Wang et al. 2007; Varner et al. 1999). Most of the available information has been gathered in the northern hemisphere. Previous studies on terrestrial ecosystems have primarily considered seven different VOCls. The most studied compounds are chloromethane (CH_3_Cl) and chloroform (CHCl_3_). Other VOCl compounds reported include CCl_4_ (tetrachlorometane), C_2_H_3_Cl (chloroethylene), CH_2_Cl_2_ (dichloromethane), CH_3_CCl_3_ (methyl chloroform) and C_2_H_3_Cl_3_ (trichloroethane) (Haselmann et al. 2002; Hoekstra et al. 2001; Rhew et al. 2008; Wang et al. 2007). In addition, other halogenated compounds such as bromomethane, iodomethane, trichlorofluoromethane (Freon-11) and dichlorodifluoromethane (Freon-12) are released from terrestrial sources (Khalil and Rasmussen 2000; Rhew et al. 2000; Varner et al. 2003).

Emissions of VOCl are likely small compared with wet and dry deposition of Cl. However, a ^36^Cl radiotracer study indirectly indicated substantial VOCl-associated release of Cl in soils corresponding to 0.18 g m^−2^ year^−1^ or 44% of the annual wet deposition (Bastviken et al. 2009). This number needs validation, but, interestingly, it includes all possible VOCls in contrast to other estimates that measure specific VOCl compounds only. Previous studies showed average Cl emission from a coniferous forest soil of 0.13 and 0.04 g m^−2^ year^−1^ released as chloroform and chloromethane, respectively (Dimmer et al. 2001), while a Cl emission of < 0.01 g m^−2^ year^−1^ was released as chloroform from a Scots pine forest soil (Hellen et al. 2006). Coastal areas and wetlands seem to be significant sources with large emissions of chloromethane at, on average 0.6 g m^−2^ year^−1^ and 0.2 g m^−2^ year^−1^ respectively (Svensson 2019). Given this, the formation of VOCl would not only represent a substantial proportion of the emission to the atmosphere but also a significant part of the chlorine cycle.

## Ecosystem Cl budgets

**Fig. 2:**
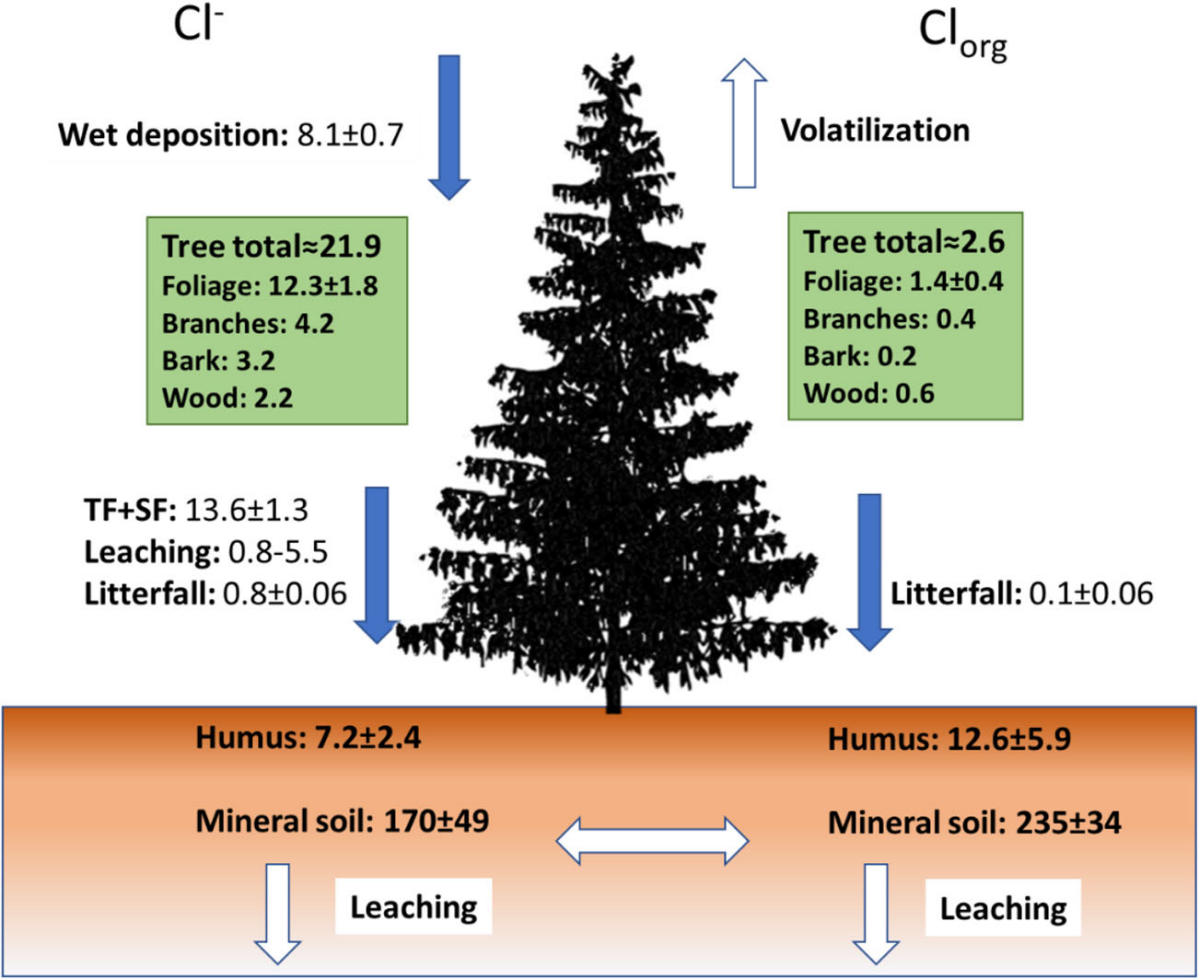
A terrestrial Cl budget of a Norway spruce forested ecosystems in France (at the experimental forest site at Breuil-Chenue, Eastern France). Pool (kg ha^−1^) and fluxes (kg ha^−1^ year^−1^). Volatilisation and soil leaching were not measured in the study (data obtained from Montelius et al. 2015)

Ecosystem budgets are typically based on concentration measurements in combination with information about carbon and water cycling that supports estimates of Cl transport and transformation. Different attempts to estimate Cl budgets (Montelius et al. 2015; Redon et al. 2011; van den Hoof and Thiry 2012; Öberg et al. 2005) all show that the order of magnitude of Cl pools is similar, though there are less data on vegetation. Figure 2 shows the current most solid Cl ecosystem budget. Further, the studies only cover temperate forest ecosystems in Northern Europe.

Net ecosystem budgets of Cl^−^, i.e. comparison of atmospheric deposition and stream export, are common because Cl^−^ is often included in monitoring efforts. A data summary indicates imbalances in many catchments (Svensson et al. 2012). This is not surprising given the new understanding of processes that either retain (plant uptake, formation of Cl_org_) or release (decay of biomass, dechlorination) Cl^−^. Imbalances were most striking in areas with a wet Cl^−^ deposition below 6 kg ha^−1^ year^−1^ suggesting unknown dechlorination/degradation processes (Svensson et al. 2012).

Lysimeter studies have specifically addressed Cl^−^ balances by irrigating soil cores with artificial precipitation with known Cl^−^ concentrations and monitoring the efflux (Bastviken et al. 2006; Rodstedth et al. 2003). These studies showed imbalances that the soil in some cases had acted as a sink and in others as a source of Cl^−^ indicating Cl transformation in topsoil. Changes in the large storage of Cl_org_ in soil were suggested to explain the pattern, and substantial leaching of Cl_org_ was observed. The soil in the experiment was, however, too heterogeneous to determine whether a change such as net loss or accumulation in the soil had taken place or not.

Cl transport through soils was previously assumed to reflect water transport in catchments. The application of Cl^−^ as a tracer is probably sufficient in areas with a large Cl^−^ deposition such as in the study by Kirchner et al. (2000), whereas studies of soils with low Cl^−^ deposition indicate retention of chloride, which may lead to a change in residence time (Bastviken et al. 2006). Thus, Cl^−^ may occasionally be a poor indicator of water retention. Few studies have addressed this issue. In a field tracer study in a small catchment, simultaneously injecting ^36^Cl and radioactive water (^3^H_2_O) into soil over a 30-day period, the recoveries in runoff of ^36^Cl and ^3^H_2_O were 47% and 78% (of the injected amount), respectively, when the tracers were injected into surface soil, while they were 83% and 98% (of the injected amount), respectively, after injections into deeper soils (Nyberg et al. 1999). Clearly, Cl^−^ is preferentially retained in topsoil. Further, overall Cl residence times, i.e. considering both Cl^−^ and Cl_org_ pools and fluxes, were five times higher than residence times based on Cl^−^ only (Redon et al. 2011). However, data have primarily been collected in forests, and to some extent also arable land, and peat bogs, while wetlands, sediments and discharge areas, where potential ^36^Cl contamination will leave underground aquifers, are poorly studied.

## Future challenges

Previous findings, primarily based on studies of bulk chlorine pools, reveal that (1) Cl^−^ is more reactive than previously thought, (2) many organisms actively produce enzymes that are involved in an extensive and rapid chlorine cycling, (3) that this cycling is linked to carbon and possibly other elements and (4) that Cl is probably much more important to, e.g. vegetation, microorganisms and carbon cycling than hitherto understood. However, several fundamental questions remain. Some of these are discussed below based on the interest to understand transport, uptake and/or exposure and residence times of Cl and ^36^Cl in terrestrial and limnic ecosystems. Cl^−^ and Cl_org_ pools will behave differently, e.g. with respect to solubility, bioavailability and residence time, and inorganic and organic Cl pools have to be considered separately in order to understand and model the dynamics of Cl in the environment over time.

### What is the ecosystem variability of Cl^−^ and Cl_org_?

This fundamental question includes inputs, standing stocks and outputs from various types of landscape units. Previous studies, generally non-repeated snapshots, have revealed differences between soil types, as well as local variations within sample plots, but only include studies in temperate regions and terrestrial ecosystems within a few different ecosystems (Johansson et al. 2003b). Currently, there are no systematic assessments of the temporal variability at different spatial scales. Such measurements are key for further studies, and for very fundamental aspects such as (1) the design of sampling programs, (2) understanding data comparability over time (seasonality) and space and (3) assessing model uncertainties. Although seemingly simple and basic, studies of ecosystems variability are fundamental to build appropriate environmental models. Soil and ground water discharge areas, e.g. wetlands, streams and lakes, are likely recipients of sub-surface sources of Cl. At present, while Cl^−^ measurements are common in limnic monitoring, there is, to our knowledge, no systematic data regarding Cl cycling of the separate aquatic Cl^−^ and Cl_org_ pools, and information on Cl cycling in aquatic systems is largely missing (Dugat-Bony et al. (2016).

### What are the main drivers of Cl^−^ and Cl_org_ accumulation and transport?

Long-term modelling with ambitions to include varying environmental conditions requires an understanding of how levels of Cl^−^ and Cl_org_ are regulated. Fundamental environmental variables such as water content, organic matter content, primary productivity, dominating vegetation, nutrient levels and temperature may have large impact on the relative distribution of Cl^−^ and Cl_org_ and affect bioavailability, transport pathways and residence times in ecosystems. In addition, a better understanding of biological chlorination would greatly facilitate modelling of chlorine cycles and for instance management of Cl accumulation in soils affected by salinisation.

Dechlorination rates, i.e. release of organically bound Cl from the organic matter, of bulk soil Cl_org_ are a poorly understood key factor for understanding terrestrial Cl cycling (Montelius et al. 2016). It is likely that some of the Cl_org_ we observe in soils represents a dynamic pool undergoing rapid turnover while some of the measured soil Cl_org_ pool may consist of very stable compounds, but the balance between these Cl_org_ pools is not clear. Therefore, rates and regulation of dechlorination are important to understand and predict the fate of Cl_org_, such as upon of land use change causing leakage or accumulation of Cl (Kauffman et al. 2003; Lovett et al. 2005; Mannerkoski et al. 2005) and potential effects due to land management such as deforestation.

In terms of transport, circumstantial evidence presented above highlight that the emissions of the total VOCl from various landscape compartments need to be quantified. Available data indicate that total VOCl emissions (in contrast to individual VOCl compounds) could be a substantial part of the export of chlorine from terrestrial ecosystems, but this remains to be confirmed.

Most questions regarding the regulation of Cl^−^ and Cl_org_ levels and transport have hitherto not been addressed and will require experimental assessments. However, other approaches may also prove valuable. For example, better understanding of the chemical composition and structure of Cl_org_ would greatly facilitate interpretations regarding chemical prerequisites for chlorination and dechlorination, what enzymes are involved and under what conditions biotic/abiotic processes may occur.

### Reaching a more comprehensive understanding of Cl cycling in terrestrial environments

As explained above, Cl cycling models need information about both Cl^−^ and Cl_org_. Presently available data for both Cl^−^ and Cl_org_ are typically based on single “snap-shot” measurement with information from a limited number of environments. These data support static mass balance models, models that are restricted by several assumptions, including the steady-state assumption, i.e. that Cl_org_ behaves as organic matter in general. Further, model uncertainties are difficult to assess given the poor knowledge of spatial and temporal variability. Also, the relatively large importance of biomass part of the total catchment Cl can be found in biomass show that many previous beliefs influencing model assumptions need to be re-evaluated.

Thus, without data covering the temporal dimension for both Cl^−^ and Cl_org_ separately, along with relevant environmental variables in sediment, water, soil and biomass pools, and without information of how important processes (e.g. chlorination and dechlorination) are regulated, long-term dynamic models of Cl-cycling largely remain guesswork. Hence, we still depend on very fundamental and partly descriptive studies of Cl in multiple environments to reach a deeper understanding about the terrestrial Cl cycling. Such studies need to consider Cl^−^ and Cl_org_ separately in as much detail as possible and should preferably be linked to simultaneous studies of water and other element cycles and biological activity at the same locations.

## Data Availability

All data generated or analysed during this study are included in this published article.
